# MiRNA: Involvement of the MAPK Pathway in Ischemic Stroke. A Promising Therapeutic Target

**DOI:** 10.3390/medicina57101053

**Published:** 2021-10-01

**Authors:** Agnese Gugliandolo, Serena Silvestro, Cinzia Sindona, Placido Bramanti, Emanuela Mazzon

**Affiliations:** IRCCS Centro Neurolesi “Bonino-Pulejo”, Via Provinciale Palermo, Contrada Casazza, 98124 Messina, Italy; agnese.gugliandolo@irccsme.it (A.G.); serena.silvestro@irccsme.it (S.S.); cinzia.sindona@irccsme.it (C.S.); placido.bramanti@irccsme.it (P.B.)

**Keywords:** ischemic stroke, miRNA, MAPK pathway

## Abstract

Ischemic stroke (IS) is a cerebrovascular disease with a high rate of disability and mortality. It is classified as the second leading cause of death that arises from the sudden occlusion of small vessels in the brain with consequent lack of oxygen and nutrients in the brain tissue. Following an acute ischemic event, the cascade of events promotes the activation of multiple signaling pathways responsible for irreversible neuronal damage. The mitogen-activated protein kinase (MAPK) signaling pathway transmits signals from the cell membrane to the nucleus in response to different stimuli, regulating proliferation, differentiation, inflammation, and apoptosis. Several lines of evidence showed that MAPK is an important regulator of ischemic and hemorrhagic cerebral vascular disease; indeed, it can impair blood–brain barrier (BBB) integrity and exacerbate neuroinflammation through the release of pro-inflammatory mediators implementing neurovascular damage after ischemic stroke. This review aims to illustrate the miRNAs involved in the regulation of MAPK in IS, in order to highlight possible targets for potential neuroprotective treatments. We also discuss some miRNAs (miR), including miR-145, miR-137, miR-493, and miR-126, that are important as they modulate processes such as apoptosis, neuroinflammation, neurogenesis, and angiogenesis through the regulation of the MAPK pathway in cerebral IS. To date, limited drug therapies are available for the treatment of IS; therefore, it is necessary to implement preclinical and clinical studies aimed at discovering novel therapeutic approaches to minimize post-stroke neurological damage.

## 1. Introduction

Stroke represents one of the main causes of mortality and morbidity in global health [1]. Two pathological subtypes of stroke can be identified, which are ischemic stroke (IS) and hemorrhagic stroke [2]. Hemorrhage is indicated by the presence of blood in the cerebral parenchyma. Cerebral strokes are predominantly ischemic and IS represents 87% of all stroke cases [3]. IS has a multifactorial etiology and is a disease of enormous impact on public health, being one of the leading cause of disability [2,4,5]. IS is induced by occlusion of small vessels in the brain and atherosclerosis affecting the cerebral circulation. These events result in the interruption of blood flow and consequently the death of the cerebral tissues. Several risk factors are known to increase the risk for IS, such as age, gender, hypertension, diabetes mellitus, hypercholesterolemia, excessive alcohol consumption, cigarette smoking, or other stressors [2,6,7,8]. IS not only alters the homeostasis of the nervous system, but also affects other tissues and organs following the genesis of atherosclerotic and inflammatory processes causing heart failure [4]. Brain tissue is extremely sensitive to changes in glucose and oxygen supply, thus a brief interruption in blood flow leads to neurological deficits.

Indeed, the brain requires about 20% of the total body oxygen consumed in resting conditions [9], and both oxygen and glucose represent the energy source for generating ATP [10].

Reperfusion means recovery of blood flow to the ischemic tissue. However, it can itself trigger a cascade of events responsible for a paradoxical injury of the tissue [11]. Ischemic injury and reperfusion are involved in several anatomical and functional alterations of the cerebrovascular system such as leakage of the blood–brain barrier (BBB), alteration of ionic homeostasis resulting in the release of glutamate and accumulation of intracellular calcium and sodium, increase in nitric oxide (NO), and apoptosis [12,13]. Cerebral ischemia compromises the BBB integrity and its disruption appears soon after the onset of artery occlusion and continues for several days to weeks after stroke [14]. The BBB disruption is induced by compromised tight junctions and endothelial damage, leading to enhanced permeability of the affected vessels [15]. The initial alteration of the BBB permeability occurs in the hyperacute stage of stroke within the first 6 h after onset in both preclinical [16,17,18] and clinical studies [19,20]. During the next 72/96 h, in the acute phase of IS, the neuroinflammation processes further lead to the disruption of the BBB [21], inducing the second peak of permeability [16,18,22]. Several lines of evidence showed that permeability remains increased up to weeks after stroke [17,22,23], suggesting that BBB stays opened during the subacute and the chronic stages of stroke. IS causes several adverse effects that trigger a cascade of events that induce neuropathic processes including oxidative stress and inflammation [24]. Indeed, various inflammatory cells are involved during ischemic brain injury, releasing both pro-inflammatory and oxidized compounds. The reperfusion event following the cerebral ischemic attack promotes the activation of biochemical processes inducing overproduction of reactive oxygen species (ROS) and reactive nitrogen species (RNS) which are decisive in inducing pathological brain damage [1]. To date, the recombinant tissue plasminogen activator is the only pharmacological compound approved for the treatment of IS, which must be administered within 4.5 h from its onset [25]. For this reason, innovative therapeutic strategies are being tested in order to discover novel therapeutic targets, to limit permanent brain damage and disability.

Although several mechanisms are involved in IS pathogenesis, different evidence demonstrates that increased expression of mitogen-activated protein kinase (MAPK) in cerebral ischemia plays a key role in the activation of inflammatory processes [26,27]. MAPKs are involved in the regulation of various biological processes such as cell proliferation, differentiation, migration, and apoptosis [28]. They include three sub-families that are c-Jun N-terminal kinase (JNK), p38 mitogen-activated protein kinases (p38), and extracellular signal-regulated kinase (ERK) 1/2. JNK is primarily activated under conditions of inflammation, stress, and stimulated by growth factors. Instead, the activation of ERK 1/2 is dependent on growth factors and cytokines, while p38 is also activated by cytokines, stress, and inflammation [29]. Furthermore, it has already been widely shown that MAPK promotes the expression of apoptotic proteins by enhancing neuronal cell death during cerebral infarction [30].

The miRNAs (miR) are small non-coding RNA (19–24 nucleotides) that play a key role in biological processes through the regulation of messenger RNA (mRNA) expression [31]. The miRNA act in post-transcriptional gene silencing, through the binding to the coding region and both to the 3′ and 5′ untranslated region of the mRNAs. The miRNAs degrade or block the target mRNA through a “hetero-silencing” mechanism [32]. Different miRNAs could regulate a single mRNA; at the same time, several mRNAs could be regulated from the same miRNA. The miRNAs are implicated in several pathological conditions (such as cardiovascular diseases, cancer, arthritis, cataracts, osteoporosis, diabetes/obesity, and hypertension) and in different neurodegenerative diseases such as Alzheimer’s disease, Huntington’s disease, Parkinson’s disease, amyotrophic lateral sclerosis, and schizophrenia [33,34,35]. Previous studies have demonstrated changes in miRNA expression profiles following IS, highlighting that miRNAs may be implicated in ischemic pathophysiology [36]. Therefore, miRNAs, through the regulation of genes linked to pathologies, could represent a system useful to maintain neuronal homeostasis permitting to respond adequately to environmental insults [37]. The purpose of this review is to illustrate the role of miRNAs implicated in the modulation of the MAPK signaling in preclinical models of IS and also to evaluate whether miRNAs in blood samples are useful as predictive and timely biomarkers of IS.

## 2. Role of miRNAs Involved in the Regulation of MAPK Pathway in IS

Recent evidence showed a role of miRNAs in the regulation of the MAPK pathway in IS pathogenesis. In recent times, several experimental studies were performed to identify the role of miRNAs in IS in order to use them as diagnostic and prognostic markers or as a potential therapeutic target.

### 2.1. The miRNAs Involved in the Regulation of Apoptosis

Upregulation of miR-195 could exert neuroprotective effects in cerebral infarction. Chang et al., suggested that miR-195 can downregulate Krueppel-like factor 5 (KLF5) blocking the JNK pathway. As a consequence, the upregulation of miR-195 enhanced synaptic plasticity, reduced apoptosis, and inhibited JNK expression and its phosphorylation, through the KLF5 downregulation. In a middle cerebral artery occlusion (MCAO) rat model, increased cerebral infarct volume and neuronal loss were observed in miR-195 ^−/−^ MCAO groups, while they decreased in miR-195 mimic MCAO and *Klf5* ^−/−^ MCAO groups compared to WT-MCAO. The results were also confirmed in vitro in oxygen–glucose deprivation (OGD)-induced rat cerebral cortex cells. Thus, miR-195 would stimulate neuronal repair and could be an important regulator against stroke-induced apoptotic processes [38].

Several studies support the involvement of miR-145 in multiple pathological conditions such as cerebral infarction. Xue et al., evaluated the role of miR-145 in neuronal stem cells (NSCs) protection through the targeting of the MAPK pathway for the treatment of IS. A simultaneous increase in miR-145, ERK, and p38 expression was observed in a time-dependent manner in NSCs. Treatment with miR-145 mimic increased ERK, p38, and their phosphorylation, suggesting that miR-145 could regulate the MAPK pathway. In parallel, cell proliferation and differentiation were induced, while apoptosis was inhibited, as suggested by reduced cleaved-caspase 3 (casp-3) levels. The miR-145 mimic group showed an increase in Nestin, neuron-specific enolase (NSE), and glial fibrillary acidic protein (GFAP) levels, respectively markers for neuro-stem cells, neurons, and astrocytes. The treatment with SB203580, a MAPK inhibitor, reversed the previous results by significantly increasing the rate of apoptosis. Interestingly, IS rats transplanted into the cerebral cortex with NSCs treated with miR-145 mimic showed a faster improvement of walking ability and neurological functions as well as neuronal regeneration [39].

The miR-339 was found to be expressed at elevated levels in MCAO samples and was also up-regulated in rat adrenal medulla-derived pheochromocytoma (PC12) cells after OGD/R treatment. In order to detect miR-339 targets, differentially expressed genes in MCAO on Gene Expression Omnibus repository were evaluated. Fibroblast growth factor 9 (FGF9) and Calcium Voltage-Gated Channel Auxiliary Subunit Gamma 2 (CACNG2) were detected and confirmed as direct targets of miR-339. Specifically, it was found that miR-339, through the inhibition of FGF9/CACNG2, induced apoptosis and modulated the MAPK pathway. Indeed, PC12 cells stimulated with OGD and transfected with miR-339 mimic showed high levels of phosphorylated p38 and JNK. Conversely, upregulation of FGF9 and CACNG2 expression reduced MAPK levels. These results indicated that miR-339 modulated MAPK activation through the inhibition of *FGF9* and *CACNG2* promoting the apoptotic process [40].

Another study showed that miR-410 exerted neuroprotective effects modulating MAPK via the tissue inhibitors of metalloproteinase 2 (TIMP2) in an IS mouse model. The increase in TIMP2 was observed in IS models. Transfection with miR-410 mimic and si-TIMP2 decreased TIMP2 and p38, ERK, and JNK protein levels. The miR-410 also promoted hippocampal neuron survival, and reduced the rate of apoptosis. Moreover, in vivo miR-410 proved to be almost neuroprotective as it decreased the volume of cerebral infarction, reduced the degeneration of hippocampal neurons, and counteracted oxidative stress, enhancing the expression of glutathione peroxidase (GSH-Px) and superoxide dismutase (SOD) in conditions of IS [41].

Another study evaluated p38α involvement in IS and its regulation. While p38α mRNA expression showed no changes, p38α protein level increased, even if not in a statistical significant manner, and decreased in the brain 2 h after MCAO compared to sham. However, the p38α level returned to levels similar to control after 4 h. These results indicated that p38α protein downregulation may depend on a posttranscriptional regulation that could be mediated by miRNAs. Bioinformatics analysis showed a predicted complementarity position in the p38α 3′UTR with miR-128-3p, which was confirmed using the luciferase reporter assay. Interestingly, the miR-128-3p level increased 60 min after MCAO, reaching a peak after 2 h, and decreased to the control level after 4 h. Further investigations showed that transfection of miR-128-3p mimic reduced p38α levels in neuroblastoma SH-SY5Y cells and in brain tissue in MCAO mice. Treatment with the miR-128-3p inhibitor demonstrated an increase in the volume of cerebral infarction. Based on the previous results, it was suggested that miR-128-3p protects against ischemia-induced cell death through the downregulation of p38α expression [42].

Wu et al., observed that miR-122 was downregulated in the serum of IS patients. Moreover, an inverse correlation between infarction size and miR-122 was found. A decrease of miR-122 expression was detected also in OGD-treated Neuro2a (N2a) cells. The miR-122 Agomir transfection into OGD-treated N2a cells increased cell viability while reduced apoptosis, decreasing the level of apoptotic and autophagic proteins such as cleaved casp-3, Bcl-2-associated X protein (Bax), Beclin-2, and Microtubule-associated protein 1A/1B-light chain 3 (LC3)-II. In parallel, an increase in S-phase neuronal cells was found, while the number of cells in G0/G1 phase was decreased. Furthermore, increased expression of the E2F1 transcription factor in IS patients had been detected, showing an inverse correlation with miR-122 expression. It was found that overexpressed E2F1 suppressed miR-122, consequently promoting Sprouty homolog 2 (*SPRY2*) expression. IS mice overexpressing E2F1 or SPRY2 showed severe neurological deficits; on the contrary, in IS mice overexpressing miR-122, a partial recovery was found. Moreover, the E2F1/miR-122/SPRY2 axis modulated the MAPK pathway during IS. Indeed, ERK and p38 phosphorylation were reduced in cells and mice overexpressing E2F1 or SPRY2, but increased in those treated with miR-122 Agomir. Given the neuroprotective effects of miR-122 upregulation, it would be interesting to further investigate the E2F1/miR-122/*SPRY2* axis for the treatment of IS [43].

The miR-298 is extensively studied in several diseases such as cancer, but is not well known for its role in IS. For this reason, its role was evaluated in both OGD/R-induced N2a cells and MCAO rats. It was found that miR-298 expression was downregulated, while nuclear factor kappa-B (NF-κB) activator (ACT1) mRNA and protein expression was upregulated in a time-dependent manner in IS models. Additionally, JNK phosphorylation was increased. The treatment with miR-298 mimic decreased p-NF-κB and phosphorylated mammalian target of rapamycin (mTOR) and increased phosphorylated JNK, B-cell lymphoma 2 (Bcl-2), and beclin1. *Act1* knockdown or miR-298 mimic in OGD/R-induced N2a cells promoted the apoptotic process as demonstrated by the increase in casp-3 levels. The miR-298 targets and negatively regulate *Act1* directly binding to the 3′UTR of its transcript. As well, in vivo, injection of miR-298 mimic in MCAO mice suppressed NF-κB and mTOR, while it increased p-JNK, Bcl-2, Beclin1, and casp-3. Furthermore, miR-298 increased neurological damage associated with an increased cerebral infarction. Thus, miR-298 overexpression aggravated ischemic damage through negative regulation of Act1/JNK/NF-kB and downstream autophagy [44].

Circular RNAs (circRNAs) are a class of non-coding RNAs formed by the process of back-splicing, by direct reverse splicing or lariat driven circularization, through the covalent joining of the 5′ and 3′ ends of the spliced RNAs. Thanks to their covalently closed structure, they are resistant to exonucleases and then they are more stable than linear mRNAs. They mainly act as miRNA sponges, attenuating or preventing mRNA translation and regulating transcription and splicing of the gene. The circRNAs may also interact with RNA-binding proteins (RBPs) [45,46,47]. Interestingly, the circ_016719/miR-29c/ Mitogen-Activated Protein Kinase Kinase 6 (Map2k6) axis could contribute to I/R-induced neuronal cell injury by stimulating the progression of IS. In the hippocampal tissues of MCAO/R mice, a reduction in miR-29c levels was observed as well as an increase in levels of both circ_016719 and Map2k6. The in vivo findings were confirmed in OGD-treated mouse hippocampal HT-22 cells. In HT-22 cells, circ_016719 knockdown increased miR-29c expression and cell proliferation, and in parallel, reduced Map2k6 expression, cell apoptosis, and autophagy. It is important to note that miR-29c inhibition suppressed the effects of circ_016719 knock down, indicating that miR-29c may have a role in the reduction of apoptosis and autophagy in cells after I/R treatment. Map2k6 overexpression reduced proliferation and increased apoptosis and autophagy of cells co-transfected with miR-29c mimics and treated with OGD/R. These results indicated that Map2k6 is a direct target of miR-29c, which exerts a protective effect in HT-22 cells subjected to OGD/R treatment. The miR-29c in turn might be sponged by circ_016719 [48].

The maternally expressed 3 (MEG3)/miR-424-5p/Semaphorin 3A (Sema3A) axis may represent an ideal therapeutic target for the management of IS. Overexpression of long non-coding RNA (lncRNA) MEG3 and Sema3A and reduced expression of miR-424-5p were observed in OGD/R-treated N2a cells. The miR-424-5p targeted and negatively regulated Sema3A. In turn, MEG3 was shown to target miR-424-5p downregulating its expression. Inhibition of MEG3 promoted miR-424-5p expression and downregulated Sema3A, as well as increased cell viability and decreased apoptotic proteins. In parallel, a reduction of the phosphorylation of JNK and p38 was also observed. This result indicated that MEG3 activated MAPK through the regulation of miR-424-5p/Sema3A. In vivo, in MCAO mice, MEG3 knockdown reduced infarct volume and phosphorylation of JNK and p38. These results suggested that MEG3 can aggravate IS, promoting apoptosis and activating MAPK, through the modulation of miR-424-5p/Sema3A [49].

The miR-221 could play a key role in neuronal cell survival after IS. Zhou et al., found that lncRNA GAS5 sponged miR-221, thus modulating the expression of apoptotic proteins. GAS5 expression in brain tissue samples taken from MCAO/R rats increased. These results were confirmed in ischemia-induced rat cortical cells and neuroblastoma B35 cells, in which increased GAS5 expression and decreased miR-221 expression were observed. GAS5 knockdown or transfection with miR-221 mimic in B35 cells inhibited the expression of apoptotic proteins such as p53 upregulated modulator of apoptosis (PUMA) and the phosphorylation of JNK and H2A histone family member X (H2AX), thus demonstrating that miR-221 overexpression suppressed apoptosis. Furthermore, bioinformatics analysis and luciferase assay further demonstrated the binding of miR-221 to GAS5 and PUMA, suggesting that GAS5 modulated PUMA sponging miR-221. Altogether, the results suggested that GAS5 increased apoptosis of neuronal cells in hypoxia conditions through miR-221/PUMA axis [50].

The role of lncRNA SNHG15 and miR-18a was evaluated in both MCAO mice and OGD-induced N2a cells. An increase in both SNHG15 and Chemokine (C-X-C motif) ligand 13 (CXCL13) expression, and also a significant decrease in miR-18a was found in MCAO mice. Moreover, the mitogen-activated protein kinase kinase (MEK)/ERK signaling pathway was inhibited while the release of pro-inflammatory cytokines such as tumor necrosis factor-alpha (TNF-α) and interleukin (IL)-1β as well as casp-3 increased. As well, in vitro, in OGD-induced N2a cells, SNHG15 and CXCL13 increased, while miR-18a decreased in association with the promotion of apoptosis and ERK inactivation. Interestingly, silencing of SNHG15 reverted these effects. OGD-induced N2a cells treated with miR-18a mimic showed a marked reduction in neuronal apoptosis. These results indicated that SNHG15 was able to bind miR-18a that in turn target CXCL13. Interestingly, ERK/MEK inhibition decreased cell viability and increased apoptosis when SNHG15 was silenced or miR-18a overexpressed [51].

Several experimental studies have shown the implication of lncRNA ANRIL in IS. Its expression has been reported to be increased in IS and was thought to induce neuroprotective effects. The study by Zhong et al., reported the expression level of ANRIL and miR-199a-5p detected in MCAO mice. Interestingly, ANRIL and miR-199a-5p increased after surgery in MCAO mice while Caveolin 1 (CAV-1) was decreased. Given that CAV-1 can regulate apoptosis, apoptotic proteins were evaluated evidencing the induction of apoptosis. Specifically, an increase in Bax and casp-3 but not in Bcl-2 levels was found, in association to a significant increase in MEK and ERK phosphorylation. Consistent with the in vivo results, increased apoptosis was found in OGD-induced N2a cells, but ANRIL overexpression promoted neuronal cell viability resulting in inhibition of the apoptotic process. CAV-1 increased after ANRIL overexpression as well as p-MEK and p-ERK levels. It was assessed that ANRIL overexpression downregulated miR199a-5p that increased after OGD treatment. Evidence demonstrated that ANRIL can bind miR-199a-5p, causing its decrease. Interestingly, inhibition of miR-199a-5p showed similar protective effects compared to ANRIL overexpression, enhancing cell viability through the CAV-1/MEK/ERK pathway. The results suggested that miR-199a-5p can directly target CAV-1, and CAV-1 protective function at least in part depends on MEK/ERK [52].

### 2.2. The miRNAs Involved in Neuroinflammation

The MAPK pathway activation could affect the progression of IS. Inhibition of p38 or ERK2 reduced cerebral infarction volume and improved cognitive functions. In parallel, pro-inflammatory cytokines such as TNF-α, IL-1, IL-6, and IL-17, the phosphorylation of ERK 1/2, p-38, and JNK decreased, together with apoptosis. The knockout of the proto-oncogene tyrosine-protein kinase (*Src*) (regulator of MAPK pathway) showed neuroprotective results similar to the previous treatment. *Src* ^−/−^ MCAO rat brain samples showed a reduction of cerebral infarction volume and improvement of neurological functions. Further investigations showed reduced miR-137 expression and simultaneous increase in Src expression in the brain tissue of MCAO rats. The luciferase assay demonstrated that *Src* was targeted by miR-137. miR-137 knockout worsened cerebral infarction, cognitive and neurological abilities of MCAO rats, as well as exacerbated the inflammatory process by activating the MAPK pathway and increasing the expression of apoptotic proteins. The upregulation of miR-137 could inhibit the inflammatory process via MAPK inhibition and alleviate neurological symptoms [4].

It was found that miR-22 may modulate the genes that provide neuroprotection; thus, its possible mechanisms and molecular targets in IS have been studied. In MCAO rats, the expression of miR-22 was found to be downregulated. Downregulation of miR-22 resulted in increased levels of pro-inflammatory cytokines such as TNF-α, IL-1β, p38 phosphorylation and overexpression of inducible nitric oxide synthase (iNOS), NF-κB, macrophage inflammatory protein (MIP), prostaglandin E2 (PGE2), and cyclooxygenase-2 (COX-2) in the IS models. It was subsequently observed that miR-22 targeted p38 by regulating its expression. Thus, upregulation of miR-22 could represent a potential candidate for protection against the neurotoxic effects caused by inflammation through the p-38 MAPK pathway inhibition [53].

Studies performed in MCAO/R rat models detected downregulation of miR-21 in ischemia/reperfusion (I/R) models. However, miR-21 overexpression was shown to promote beneficial effects such as reduction in the volume of cerebral infarction, preservation of BBB integrity and reduction of edema. Furthermore, miR-21 targeted the MAPK pathway by negatively modulating Mitogen-Activated Protein Kinase Kinase 3 (*MAP2K3*). Treatment with miR-21 mimic and SB203580 (MAPK inhibitor) induced overexpression of miR-21 which consequently reduced the levels of MAP2K3, p38, iNOS, and metalloproteinase (MMP)-9, markers of neuroinflammation that were increased in the I/R conditions. Therefore, the overexpression of miR-21 would aim to preserve the permeability of the BBB through downregulation of MMP-9, thus avoiding further post-stroke neurological damage [54].

Emerging studies are focusing on stem cell-derived extracellular vesicles (EVs) in the treatment of cerebrovascular diseases such as stroke. MCAO/R mice were treated with EV of neural progenitor cells derived from the ventral midbrain mesencephalon region of the human fetal brain (ReN). In order to have a more specificity of interaction with the brain region involved in ischemic damage, the arginine–glycine–aspartic acid (RGD)-4C peptide was conjugated on the surface of EV_ReN_. Therefore, it was shown that RGD-EV_ReN_ treatment in MCAO/R mice had a tropism for integrin αvβ3 which is expressed on endothelial cells in the process of angiogenesis under conditions of IS. This treatment showed anti-inflammatory properties by reducing the release of pro-inflammatory cytokines such as IL -1β, IL-6, and TNF-α, which attenuate microglial activation. In order to better understand the mechanism of anti-inflammatory action, the differential expression of miRNA was evaluated. The miRNA sequencing has revealed seven up-regulated miRNAs in EV_ReN_ including let-7b-5p, let-7g-5p, let-7i-5p, miR-21 -5p, miR-98-5p, miR-99a-5p, and miR-139-5p, which inhibited the activation of the MAPK pathway, especially inhibited p38 phosphorylation. EV_ReN_ would exert miRNA-mediated anti-inflammatory action, which would suppress the MAPK pathway. Intravenous administration of EVs could be a targeted therapeutic strategy to optimize the treatment of IS [55].

The preclinical studies regarding miRNAs involved in the regulation of apoptosis and neuroinflammation in IS are summarized in Table 1.

### 2.3. The miRNAs Involved in the Regulation of the Post-Stroke Angiogenesis

After an ischemic event, endothelial cells are activated in the ischemic penumbra promoting the release of different pro-angiogenic factors in order to repair neuronal and vascular damage [56]. For this reason, there is a growing interest in identifying mechanisms that stimulate cerebral angiogenic processes.

**Table 1:** Experimental models of miRNAs involved in MAPK pathway linked to IS.

miRNAs	Target mRNA	Models	Experimental Outcomes	Ref.
miR-195	KLF5	MCAO-induced miR-195 knockout and *Klf5* knockout SD male rats; OGD-induced rat cerebral cortex cells	The upregulation of miR-195 enhanced synaptic plasticity, reduced apoptosis and inhibited JNK expression and its phosphorylation, through the KLF5 downregulation. The results were also confirmed in vitro in OGD-induced rat cerebral cortex cells	[38]
miR-145	-	MCAO-induced adult male SD rats; NSCs	The miR-145 overexpression promoted an increase in ERK and p38 levels in NSCs. It also induced an increase in the levels of Nestin, NSE, and GFAP, while it reduced the level of cleaved casp-3. In MCAO rats improved neurological functions. The miR-145 promoted cell proliferation through the MAPK pathway.	[39]
miR-339	FGF9 CACNG2	OGD/R induced-PC12 cells	The miR-339 promoted the phosphorylation of p38 and JNK through the inhibition of FGF9 and CACNG2.	[40]
miR-410	TIMP2	MCAO-induced male Kunming mice; Hippocampal neurons	Transfection with miR-410 mimic and si-TIMP2 increasing p38, ERK, and JNK levels in hippocampal neurons. In addition, it reduced the volume of cerebral infarction.	[41]
miR-128-3p	p38α	MCAO-induced male BALB/c mice; SH-SY5Y cells	Transfection with miR-128-3p mimic reduced p38 levels. It also exerted neuroprotective effects such as neurological improvements and reduced cerebral ischemic area.	[42]
miR-122	SPRY2	MCAO-induced male C57BL/6 mice; OGD/R induced-N2a cells	Transfection with miR-122 Agomir into N2a cells increased cell viability and reduced the levels of apoptotic and autophagic proteins such as casp-3, Bax, LC3B-II and Beclin-2 mediating neuroprotective effects against cerebral infarction.	[43]
miR-298	ACT1	MCAO-induced male C57BL/6 mice; OGD/R induced-N2a cells	Downregulation of miR-298 and increased ACT1 expression was found in IS models. Instead, injection of miR-298 mimic in MCAO rats suppressed NF-κB and mTOR, while it increased p-JNK, Bcl-2, Beclin1, and casp-3. Thus, miR-298 overexpression worsens neurological damage through negative regulation of Act1/JNK/NF-κB in IS.	[44]
miR-29c	MAP2K6	MCAO-induced male C57BL/6J mice; OGD-induced HT-22 cell	Circ_016719 sponged miR-29c that in turn target Map2k6. The downregulation of miR-29c exacerbated neuronal damage in IS models.	[48]
miR-424-5p	Sema3A	MCAO-induced male C57BL/6J mice; OGD/R induced-N2a cells	The miR-424-5p downregulated Sema3A resulting in a reduction of apoptosis, p-p38, and p-JNK levels in IS models. Furthermore, miR-424-5p reduced the volume of cerebral infarction in MCAO mice.	[49]
miR-221	PUMA	MCAO-induced adult male SD rats; primary rat cortical cell and B35 cells subjected to hypoxia treatment	The miR-221 was downregulated by lncRNA GAS5 after IS. Transfection with sh-GAS5 or with miR-221 mimic in B35 cells reduced PUMA, p-JNK and H2AX levels suppressing apoptosis.	[50]
miR-18a	CXCL13	MCAO-induced adult male C57BL/6J mice; OGD/R induced-N2a cells	The miR-18a was downregulated while CXCL13 levels increased in both MCAO rats and N2a cells. MEK/ERK pathway was inhibited increasing neuroinflammation and apoptosis as well as TNF-α, IL-1β and casp-3 levels, exacerbating neuronal damage.	[51]
miR-199a-5p	CAV1	MCAO-induced adult male C57BL/6J mice; OGD/R induced-N2a cells	LncRNA ANRIL overexpression downregulated miR-199a-5p activating CAV-1/MEK/ERK signaling. The increase in CAV1 both in vivo and in vitro exerted neuroprotective effects.	[52]
miR-137	Src	MCAO-induced miR-137 ^−/−^ C57BL/6J male mice, *Src* ^−/−^ C57BL/6J male mice, and WT C57BL/6J male mice	The miR-137 downregulated *Src* reducing cerebral infarction volume by inhibiting release of inflammatory markers such as TNF-α, IL-1, IL-6 and IL-17, p-ERK 1/2, p-38, and p-JNK and also improving cognitive functions in MCAO rats.	[4]
miR-22	p38	MCAO-induced adult male SD rats; OGD/R induced-PC12 cells	Downregulation of miR-22 increased p-38 resulting in inflammatory process via p-38 phosphorylation and increased TNF-α, IL-1β, MIP, PGE2, COX2, and iNOS levels. Therefore upregulation can exert an anti-inflammatory action useful for the prevention and treatment of IS.	[53]
miR-21	MAP2K3	MCAO-induced male SD rats	The miR-21mimic treatment inhibited MAP2K3 as well as significantly decreased p38, iNOS thereby alleviating neuroinflammation in MCAO/R rats. In addition, the latter suppressed MMP-9, preserving the integrity and permeability of the BBB.	[54]
let 7b-5p let 7g-5p let 7i-5p miR-21-5p miR-98-5p miR-99a-5p miR-139-5p	-	MCAO-induced male C57BL/6J mice; ReN cells, BV2 microglia and HEK293T cells	Seven up-regulated miRNAs were detected in EV_ReN_ as let-7b-5p, let-7g-5p, let-7i-5p, miR-21-5p, miR-98-5p, miR-99a-5p, and miR-139-5p, suppressing inflammation via MAPK pathway inhibition. Furthermore, RGD-EV_ReN_ treatment in MCAO rats reduced the release of pro-inflammatory cytokines such as TNF-α, IL -1β, and IL-6 by attenuating the activation of microglia, responsible for the exacerbation of neuroinflammation.	[55]
miR-15a miR-16-1	-	EC-miR-15a/16-1 cKO male C57BL6/J mice subjected to MCAO	Endothelial deletion of the miR-15a/16-1 cluster promoted signaling activation VEGFA-VEGFR2 and FGF2-FGFR1. In this way, the deletion of this miR cluster induces neurological recovery after IS.	[57]
miR-199b-3p	EGR1	MCAO-induced BALB/c mice; CMECs extracted from MCAO-R mice.	Stroke in MCAO rats induced miR-199b-3p downregulation and simultaneous increase in p-38, EGR-1, ERK, and Bax levels through activation of MAPK/ERK/EGR1 signaling. These results were reversed by upregulation of miR-199b-3p.	[58]
miR-126	PTPN9	MCAO-induced ICR mice; HUVEC cells	The miR-126 was downregulated in MCAO rats, therefore its overexpression induced significant improvements such as reduction of ischemic area in the brain and restoration of neurological functions. Furthermore, miR-126 targeted PTPN9 and increased p-Akt and p-ERK by promoting angiogenesis and neurogenesis.	[59]
miR-493	MIF	MCAO-induced male SD rats; OGD-induced BMECs	Downregulation of miR-493 increased MIF expression and the levels of p-Akt and p-ERK in both BMECs and MCAO rats.	[60]
miR-26a	HIF-1a	MCAO-induced male SD rats; OGD-induced BMECs;	Increasing expression of miR-26a was detected in MCAO mice after cerebral infarction as well as in OGD-induced BMECs. miR-26a was shown to increase p-Akt and p-ERK levels, promoting the proliferation and angiogenesis of BMECs after cerebral infarction.	[61]
miR-381-3p	Map3k8Cebpb	MCAO-induced BALB/c mice; EPCs were isolated from mouse bone marrow	Downregulation of miR-381-3p was found which induced an increase in Map3k8 and Cebpb in MCAO rats. Consequently, IL-6 and IL-18 levels increased while reducing angiogenesis proteins. On the other hand, the inhibition of Map3k8 and Cebpb in EPCs has been shown to reduce the inflammatory process and also the phosphorylation of p38, JNK and ERK 1/2. The upregulation of miR-381-3p could promote angiogenesis and reduce inflammation through suppressing Map3k8 and Cebpb levels.	[62]
miR-145	-	Mouse EPCs	Transfection with miR-145 in EPCs stimulated the activation of the JNK pathway promoting cell proliferation and migration. Indeed, the administration of miR-145 transfected EPCs in mouse promoted the recanalization process of arterial thrombosis after IS.	[63]

KLF5: Krueppel-like factor 5; MCAO: middle cerebral artery occlusion; OGD: oxygen-glucose deprivation; SD: Sprague Dawley; NSCs: neuronal stem cells; p38: p38 mitogen-activated protein kinases; IS: Ischemic Stroke; JNK: Jun N-terminal kinase; ERK: extracellular signal-regulated kinase; NSE; neuron-specific enolase; GFAP: glial fibrillary acidic protein; MAPK: mitogen-activated protein kinase; FGF9: Fibroblast growth factor 9; CACNG2: Calcium Voltage-Gated Channel Auxiliary Subunit Gamma 2; TIMP2: tissue inhibitors of metalloproteinase 2; SPRY2: Sprouty homolog 2; Bax: Bcl-2-associated X protein; casp-3: caspase-3; LC3:Microtubule-associated protein 1A/1B-light chain 3; NF-κB: nuclear factor kappa beta; mTOR: mammalian target of rapamycin; Bcl-2: B-cell lymphoma 2; ACT1: NF-κB activator; MAP2K6: Mitogen-Activated Protein Kinase Kinase 6; LncRNA: long non-coding RNA; Sema3A: Semaphorin 3A; PUMA: p53 upregulated modulator of apoptosis; H2AX: H2A histone family member X; CXCL13: Chemokine (C-X-C motif) ligand 13; MEK: Mitogen-activated protein kinase kinase; TNF-α: tumor necrosis factor alpha; IL: interleukin; CAV1: Caveolin 1; Src: proto-oncogene tyrosine-protein kinase; MIP: macrophage inflammatory protein; PGE2: prostaglandin E2; COX2: cyclooxygenase-2; iNOS: inducible nitric oxide synthase; MAP2K3: Mitogen-Activated Protein Kinase Kinase 3; MMP-9: metalloproteinase-9; BBB: Blood brain barrier; RGD: arginine-glycine-aspartic acid; EV: extracellular vesicle; ReN: human fetal brain; EC: Endothelial cell; VEGFA: Vascular endothelial growth factorA; VEGFR2: vascular endothelial growth factor receptor 2; FGF2: fibroblast growth factor 2; FGFR1: fibroblast growth factor receptor 1; EGR1: Early Growth Response 1; EC-miR-15a/16-1 cKO: endothelium-targeted conditional knockout miR-15a/16-1 mice; CMEC: cerebral microvascular endothelial cell; PTPN9: phosphatase non-receptor type 9; HUVEC: human umbilical vein endothelial cell; Akt: activating protein kinase; MIF: cytokine macrophage migration inhibitory factor; BMEC: brain microvascular endothelial cells; HIF-1a: hypoxia-inducible factor 1-alpha; MAP3K8: Mitogen-Activated Protein Kinase Kinase Kinase 8; Cebpb: CCAAT Enhancer Binding Protein Beta; EPC: endothelial progenitor cell.

This biological process is tightly regulated by specific miRNAs, including the miR-15a/16-1 cluster. As already known, miR-15a/16-1 expression is strongly increased in IS patients. Upregulation of miR-15a and miR-16-1 have been shown to inhibit the angiogenesis process. Sun et al., in the MCAO model, evaluated this process using endothelium-targeted conditional knockout of miR-15a/16-1 mice (EC-miR-15a/16-1 cKO). It was observed that the deletion targeted to the endothelium of the miR-15a/16-1 cluster increased the expression of angiogenesis promoters such as Vascular endothelial growth factor (VEGF)-A, fibroblast growth factor 2 (FGF2), vascular endothelial growth factor receptor 2 (VEGFR2), and fibroblast growth factor receptor 1 (FGFR1). Furthermore, increased levels of phosphorylated Src protein were observed. AZD0530-treatment, a Src inhibitor, confirmed the reduction of phosphorylated Src levels in EC-miR-15a/16-1 KO mice. AZD0530 inhibited the Src signaling pathway by reducing phospho-Src and its downstream mediators such as activating protein kinase (Akt) B, Stat3, p38 MAPK, p44/42 MAPK, and Focal adhesion kinase (FAK). Additionally, behavioral tests were performed on EC-miR-15a/16-1 cKO mice showing neurological improvements after IS. Hence, deletion of the miR-15a/16-1 cluster could be desirable as a future approach for promoting angiogenesis and improving neurological outcomes in IS [57].

Yong et al., examined the role of miR-199b-3p in the MCAO-reperfusion (MCAO-R) mice model. Mice were used to measure the expression of miR-199b-3p and the MAPK/ERK/early growth response 1 (EGR1) axis-related genes. Cerebral microvascular endothelial cells (CMECs) were extracted from the MCAO-R mice and were treated with mimic or inhibitor for miR-199b-3p, or U0126 (an inhibitor for the MAPK/ERK/EGR1 axis). The results showed downregulation of miR-199b-3p in tissues and CMECs. Conversely, it was observed an increase of levels of p38 protein, EGR1, and ERK, and an increase of apoptosis decreasing Bax levels. In line with that observation, CMECs transfected with miR-199b-3p mimics or U0126 manifested an increase of cell viability, more cells arrested at the S stage, and a reduction of apoptosis. Therefore, increased expression of miR-199b-3p could protect mice against IS, inducing proliferation through MAPK/ERK/EGR1 axis suppression [58].

Qu, M. et al., showed that miR-126-3p and miR-126-5p overexpression in human umbilical vein endothelial cells (HUVECs) induced their proliferation, migration, and tube formation. Subsequently, the role of miR-126-3p and miR-126-5p in IS was assessed in MCAO rats. Moreover, miR-126 was administered via a lentiviral vector into the brain of rats. Overexpression of miR-126, specifically in the striatum and cortex, resulted of improvements in impaired neurological functions. Additionally, the increased expression of this miRNA activated Akt and ERK pathways and their downstream genes and reduced the expression of Tyrosine-protein phosphatase non-receptor type 9 (PTPN9) in neurons. PTPN9 is involved in inhibiting angiogenesis by downregulating Akt and MAPK. In this way, miR-126 overexpression could be useful in post-stroke recovery as it would stimulate the restoration of brain functions through the stimulation of angiogenesis and neurogenesis [59].

The miR-493 was found downregulated in IS patients. The downregulation of this miRNA was detected in both OGD-induced brain microvascular endothelial cells (BMECs) and the ischemic boundary zone (IBZ) of MCAO rats. It was performed an injection of agomir-493 (a synthetic agonist of miR-493) into the rat brain increased miR-493 levels and reduced capillary density. BMECs were transfected with miR-493 mimic or with miR-493 inhibitor. Inhibition of miR-493 has shown promising results by promoting both capillary-like tube formation and endothelial cell migration. The possible targets of miR-493 were examined, in order to evaluate its correlation with genes involved in the angiogenesis process, including cytokine macrophage migration inhibitory factor (MIF). MIF in both MCAO rats and in OGD-induced BMECs was upregulated. Further investigations revealed that transfection with miR-493 mimic into OGD-induced BMEC reduced MIF levels, whereas the reverse results were obtained by transfection with miR-493 inhibitor. These data demonstrated that MIF was a target of miR-493. Additionally, the miR-493 inhibitor increased phosphorylated Akt and ERK levels. Moreover, miR-493 downregulation promoted angiogenesis, reducing ischemic damage and improving neurological deficits [60].

Liang et al., assessed the specific role of miRNA-26a in a rat model of IS and its underlying mechanism. It was shown an increased expression of miR-26a as early as the following day of cerebral infarction induction in MCAO mice. Similarly, miR-26a was seen to be upregulated in OGD-induced BMECs. To better understand the role of miR-26a BMECs were transfected first with miR-26 mimic and then with miR-26a inhibitor. Transfection with miR-26a mimic was found to promote proliferation compared to inhibitor treatment. miR-26a was shown to be involved in the phosphorylation of Akt and ERK 1/2. In this way, miR-26a might up-regulate the expression of hypoxia-inducible factor 1-alpha (HIF-1a) via the activation of Akt and ERK1/2 pathways. Thus, this miRNA could mediate the transcriptional activity of VEGF promoting lumen formation, cell proliferation, and endothelial angiogenesis in BMECs after cerebral infarction [61].

Li et al., assessed the potential role of miR-381-3p in MCAO rats and its underlying mechanism. It was demonstrated that the downregulation of miR-381-3p was responsible for the increase in Mitogen-Activated Protein Kinase Kinase Kinase 8 (Map3k8) and CCAAT Enhancer Binding Protein Beta (Cebpb) in IS, with the consequent implication of the TNF-α signaling pathway. Immunostaining of brain tissues revealed Map3k8 cytoplasmic and Cebpb nuclear localization and a marked increase in the volume of cerebral infarction. Transfection with sh-Map3k8 and sh-Cebpb in endothelial progenitor cells (EPCs) resulted in an increase in angiogenesis proteins such as VEGF, basic fibroblast growth factor (bFGF), Platelet-derived growth factor subunit B (PDGF-β) and insulin-like growth factor (IGF-1) and a significant decrease in IL-1β, IL-6, IL-18, and TNF-α. Furthermore, the silencing Map3k8 or Cebpb inhibited the phosphorylation of P38, JNK, and ERK1/2. Thus, it is possible to define that upregulation of miR-381-3p would suppress the levels of Map3k8 and Cebpb promoting angiogenesis and reducing inflammation [62].

Chen et al., investigated the role of miR-145 in the regulation of EPCs useful in the recanalization of arterial thrombosis after cerebral infarction. Transfection with miR-145 into EPC has been shown to promote cell growth, migration, and not stimulate the apoptotic process. Transfection with miR-145 stimulated activation of the JNK pathway. Indeed, treatment with SP600125 (JNK inhibitor) significantly reduced JNK levels as well as inhibited cell proliferation and migration. To confirm the results obtained in vitro, an intravenous injection of EPC transfected with miR-145 was performed in cerebral infarction rats, thus promoting the process of recanalization of arterial thrombosis [63].

The primary purpose of post-stroke angiogenesis is to generate a network of new blood vessels to repair and protect against ischemic damage. Growing evidences suggest that both endothelial cells and miRNA expression can promote post-stroke angiogenesis via the MAPK pathway (Table 1). However, there are not many reports regarding this regulatory mechanism, but further investigation would be needed to implement knowledge of the role of miRNAs in post-stroke angiogenesis

## 3. The miRNA as Potential Predictive Biomarker of IS

IS cannot be identified based only on clinical assessment, thus a rapid diagnosis remains a challenge. Computed tomography and magnetic resonance imaging are useful tools for the diagnosis of stroke [64]. It would be valuable to discover non-invasive tests that aim to quickly identify IS from stroke mimics or hemorrhagic stroke. Therefore, early diagnosis and new markers are necessary for prompt therapeutic intervention. Until today, among biomarkers identified, none so far have shown sufficient sensitivity and specificity to be used in the clinical field. Circulating miRNAs are known as suitable biomarkers in several pathological conditions. These biomarkers are non-invasive and are fast-sensing. Different findings have shown that miRNAs could predict several cellular and molecular pathways implicated in IS [65,66]. It was revealed that miRNAs are implicated in the different stages of IS and in stroke recovery [65,66,67,68]. Hence, identification of miRNAs may represent new biomarkers and therapeutic targets for IS.

However, a limitation of the use of plasma or serum miRNAs is due to the extremely low concentration of miRNAs circulating in these biological fluids. In vivo microarray studies have shown that the concentration of numerous miRNAs changes following an ischemic event. In a study performed by Huang S. et al., it was shown that the blood level of let-7e-5p was significantly increased in patients with IS compared to control patients. It was also observed that the increase in the concentration of miRNAs was directly related to a higher incidence of IS. Furthermore, bioinformatic analysis and investigation of target gene expression have highlighted the role of this miRNA in the pathogenesis of IS. In vitro studies confirmed that transfection of cells with let-7e-5p mimics decreased the expression of casp3 and nemo-like kinase (NLK), a downregulation that was also observed in whole blood samples from IS patients compared to healthy subjects [69].

In another study, it was shown that miRNA-128 was overexpressed in the peripheral blood of IS patients and the increase in this miRNA was directly correlated with the progression of damage. Additionally, bioinformatic analyses highlighted the involvement of miR-128 in the MAPK signaling pathway and in cell cycle progression. In vitro investigations also confirmed that treatment of primary neurons with miR-128 antagomir exacerbated neuronal damage-inducing cell cycle activation. Therefore, these data encourage the use of this miRNA as a potential biomarker in the diagnosis of IS [70].

A group of researchers created a serum miRNA expression profile in middle-aged IS patients. In this survey, 117 acute IS patients and 82 healthy subjects were recruited. The analysis revealed a change in the expression profile of 115 miRNAs under pathological conditions. Based on the results, miR-106b-5p, miR-532-5p, and miR-1246 could be involved in the progression of this cerebrovascular disease and could modulate different pathways including MAPK. According to the literature, a change in the miR-1246 expression profile has already been detected in blood samples from IS patients [71]. In conclusion, from these findings, it is inferred that different miRNAs altered following ischemic damage can modulate different signaling pathways involved in IS or promote the regeneration of damaged tissue. Therefore, many of these miRNAs could be potential biomarkers for prediction or early diagnosis. However, since the function of circulating miRNAs is often correlated with each other, the identification of miRNA panels could ensure greater sensitivity and specificity in the diagnosis [72,73]. Furthermore, further studies are needed to analyze the biological consequences that the expression of these miRNAs can have in cerebrovascular diseases. Prospective cohort studies are needed to evaluate the random relationship between the change in expression of these miRNAs and the onset of the disease.

## 4. Conclusions

This review provided an overview of the studies that demonstrated the involvement of miRNAs in the modulation of the MAPK pathway in IS. The evidence indicated that some miRNA are dysregulated in IS, influencing its pathogenesis through an altered regulation of the MAPK pathway. These miRNAs can modulate processes occurring after stroke such as inflammation, oxidative stress, and cell death. Specifically, some miRNAs, such as miR-145, miR-137, miR-493, and miR-126, act as regulators of mechanisms of inflammation, apoptosis, angiogenesis, and neurogenesis through the modulation of the MAPK pathway. The regulation of these miRNAs’ expression could limit permanent neuronal damage, through the modulation of several target genes involved in IS. However, there are limitations in the use of miRNAs in the medical field due to their ability to simultaneously regulate many target genes and their unclear molecular mechanism. Furthermore, some circulating miRNAs involved in the MAPK pathway regulation may represent predictive and useful biomarkers to enable early diagnosis of IS, even if they present some limitations, such as the low concentration. The modulation of the MAPK pathway by miRNA showed promising results in preclinical models. However, further studies are needed to provide miRNA therapies applicable in clinical practice for the IS management.

## Data Availability

Not applicable.
